# Visual prediction of outcomes in patients undergoing intravenous thrombolysis

**DOI:** 10.1371/journal.pone.0336226

**Published:** 2025-12-18

**Authors:** Qing Liang, Tao Qie, Yinglei Li

**Affiliations:** Department of Emergency Medicine, Baoding NO.1 Central Hospital, Baoding, China; Fondazione Policlinico Universitario Agostino Gemelli IRCCS, ITALY

## Abstract

**Background:**

This research presents a novel visual predictive model aimed at the early identification of patients at elevated risk of poor prognosis following intravenous thrombolysis, assessed six months post-acute ischemic stroke.

**Methods:**

A retrospective cohort of patients who underwent intravenous thrombolysis at advanced stroke centers was analyzed. The latest Least Absolute Shrinkage and Selection Operator (LASSO) regression technique was employed to select relevant variables and develop nomograms. The model’s performance was evaluated through receiver operating characteristic (ROC) curves, calibration curves, and decision curve analysis, culminating in an assessment of the model’s reliability.

**Results:**

We identified five principal predictors that are significantly associated with a 6-month adverse prognosis in patients undergoing intravenous thrombolysis. These predictors include door-to-needle time (DNT), homocysteine (HCY) levels, lactate dehydrogenase (LDH) levels, the post-thrombolysis National Institutes of Health Stroke Scale (NIHSS) score (P-NIHSS), and the monocyte to high-density lipoprotein cholesterol (MHR) ratio. The nomogram’s AUC-ROC was 0.914 (95% CI: 0.899–0.939) for the training cohort and 0.892 (95% CI: 0.852–0.932) for the validation cohort.

**Conclusion:**

This straightforward visual prediction model effectively identifies factors linked to poor prognosis 6 months post-intravenous thrombolytic therapy for acute ischemic stroke, aiding early treatment and resource allocation.

## Introduction

Stroke is a cerebrovascular disease with a high mortality rate, divided into haemorrhagic and ischaemic strokes [1,2]. Ischaemic stroke is the most common cerebrovascular disease and has a major impact on health, with increased morbidity and mortality. With the publication of the results of the National Institute of Neurological Disorders and Stroke (NINDS) rt-PA stroke trial in 1995 [3,4], intravenous thrombolysis (IVT) became the primary treatment for acute ischaemic stroke (AIS). Thrombolysis can effectively relieve ischaemic haemiphrenia and improve cerebral ischaemia and hypoxia, but it can cause serious complications such as reperfusion injury and cerebral haemorrhage. Therefore, early identification of risk factors for poor prognosis after thrombolysis is critical, as is timely intervention, management and implementation of rehabilitation programmes. There is a lack of comprehensive research on the long-term outcomes, quality of life and factors influencing thrombolysis in patients who receive thrombolysis after the initial period of hospitalization [5]. Studies have shown that the typical recovery period for stroke patients is three to six months, during which time sequelae may occur. In addition, studies have shown an association between functional status at 6 months after ischaemic stroke and long-term survival [6]. It is therefore very important to monitor the 6-month prognosis of patients with acute ischaemic stroke. Given the diversity and variation in clinical indicators for these patients, clinicians face a heavy workload. And it predicts the prognosis of patients for a longer period of 6 months. The main focus of current research is therefore to develop rapid, efficient, direct and accurate methods to assess patient outcomes. In addition, translating clinical data into visually intuitive and user-friendly tools is a major challenge.

In this study, we used minimum absolute contraction and selection operator regression methods to develop simplified visualisations using clinical features and laboratory results as variables to identify the variables most associated with prognosis. These nomograms [7] allow scores to be calculated based on individual patient characteristics to predict the likelihood of poor prognosis in patients with acute ischaemic stroke at 6 months after thrombolysis, providing an early personalised prognostic assessment for patients with acute ischaemic stroke (AIS) undergoing intravenous thrombolysis.

## Materials and methods

### Research subjects

Our data came from a retrospective study of Baoding First Central Hospital (National Advanced Stroke Centre) from January 2017 to February 2023. Data were used for research purposes from 07/05/2023. This study was conducted in accordance with the tenets of the Declaration of Helsinki and approved by the Ethics Committee of our Institute (Ethics Batch Number: 2023−017). This was a retrospective study and, following approval by the hospital ethics review board, patient consent was not required and all data were anonymised to ensure confidentiality. Inclusion criteria: [1] age ≥ 18 years; [2] patients fulfilling the indications for intravenous thrombolysis within 4.5 hours of the onset of acute ischaemic stroke and treated with a standard dose of alteplase (0.9 mg/kg) [8]; [3] obtaining informed consent from patients and their families. Exclusion criteria: [1] patients with a history of stroke before the current episode and mRS score ≥2; [2] patients who received endovascular therapy; [3] death within 24 hours after thrombolysis; [4] patients diagnosed with stroke-like illness after admission; [5] patients with incomplete test data; [6] patients lost to follow-up.

### Data collection

Data from patients with acute ischaemic stroke who received intravenous thrombolytic therapy with alteplase were retrospectively collected and randomised into two groups according to the 7:3 principle: the experimental group, the training group and validation group. There was no difference in the distribution of clinical data between the two groups. Demographic characteristics, vascular risk factors, laboratory indicators, individual patient information, clinical examination results and follow-up information were collected. The location of stroke lesions is classified as anterior or posterior [9]. According to the TOAST classification [10], the aetiological classification of ischaemic stroke, stroke mainly includes large atherosclerotic stroke, cardiac embolism, small-artery occlusion or lacunar stroke, as well as ischaemic stroke and idiopathic ischaemic stroke caused by other causes.

Clinical examination data collected included pre-baseline NIHSS score (i.e., pre-thrombolytic NIHSS score, ANIHSS score), post-thrombolytic NIHSS score (i.e., post-thrombolytic NIHSS score, PNIHSS score), baseline systolic blood pressure (SBP), baseline diastolic blood pressure (DBP), time from onset to treatment (OTT), and door-to-needle time (DNT).

Vascular risk factors include hypertension, diabetes, coronary heart disease (CHD), hyperlipidaemia, smoking (current or former), hyperhomocysteinemia (HCY) and atrial fibrillation. Hypertension was defined as previously diagnosed hypertension, oral antihypertensive medication or post-hospital blood pressure ≥140/90 mmHg; diabetes is associated with previous use of hypoglycemic agents or diabetes diagnosed during hospitalization; coronary heart disease is defined as coronary heart disease diagnosed in the past or at the time of admission; hyperlipidaemia was defined as previous use of lipid-lowering medication or total cholesterol ≥5.18 mmol/L, triglycerides ≥1.70 mmol/L, low-density lipoprotein cholesterol ≥3.37 mmol/L or high-density lipoprotein cholesterol ≤1.04 mmol/L during hospitalization; atrial fibrillation refers to a previous diagnosis of atrial fibrillation or an electrocardiogram showing atrial fibrillation after admission; Smoking includes current or former smoking, defined as continuous or cumulative smoking of at least one cigarette a day for six months or more over a lifetime; hyperhomocysteinemia was defined as a total blood homocysteine level ≥10 μmol/l.

Laboratory results collected at enrolment included baseline blood glucose (Glu), white blood cell (WBC) count, haemoglobin (HB) count, platelet count (PLT), neutrophil (N) count, lymphocyte (L) count, monocyte (M) count, eosinophil (E) count, neutrophils/lymphocytes (NLR) and platelet/neutrophils (PNR), monocyte/neutrophils (MNR), neutrophils/eosinophils (NER), eosinophils/monocytes (EMR), prothrombin time (PT) and partial activated prothrombin time (APTT). Other laboratory tests included blood homocysteine (HCY), lactate dehydrogenase (LDH), brain natriuretic peptide (BNP), creatinine (CR), uric acid (UA), total cholesterol (TC), triglycerides (TG), high-density lipoprotein cholesterol (HDL), low-density lipoprotein cholesterol (LDL), high-density lipoprotein cholesterol and low-density lipoprotein cholesterol ratio, ratio of high-density lipoprotein cholesterol to low-density lipoprotein cholesterol, ratio of monocytes to macrophages, low-density lipoprotein cholesterol (LDL), ratio of high-density lipoprotein cholesterol to low-density lipoprotein cholesterol (HDL/LDL), ratio of monocytes to high-density lipoprotein cholesterol (MHR).

### Outcome measures

We used a 6-month modified Rankin Scale (mRS) score [11] to assess the patients’ condition. Patient clinical outcomes were defined as poor prognosis with an mRS score of 3–6 and good prognosis with an mRS score of 0–2. Observations were assessed by a senior doctor in return telephone calls or face-to-face meetings, and disagreements were adjudicated by a third doctor.

### Statistical methods

R statistical software (version 4.0.2) was used for statistical analysis of the outcome data. The Kolmogorov-Smirnov goodness of fit test was used for continuous variables, normal distribution variables were expressed as mean ± standard deviation, and abnormal distribution variables were expressed as median and interquartile range. Categorical variables are expressed as frequencies and percentages (%). All data were entered into the R system and randomly divided into a training group and a validation group in a 7:3 ratio [12]. The initial variables [13] were screened using LASSO regression and relevant predictors were selected to minimise model over-fitting. The variables screened by LASSO regression analysis were included in the subsequent multivariate logistic regression analysis. In multivariate logistic regression analysis, variables with *P* < 0.05 were considered statistically significant. A nomogram was constructed using the results of the binary logistic regression model and the validity of the nomogram was assessed by the validation cohort. The performance of the graph is assessed by calculating the area under the receiver operating characteristic curve (ROC). The accuracy of the model is assessed by correcting the graph and comparing the predicted probability of the model with the actual results. We evaluated the clinical effectiveness of the model using decision curve analysis and probability net benefit analysis, and finally analysed the rationality of the model. Significance was set at *P* < 0.05.

## Results

### Baseline characteristics

**Fig 1:**
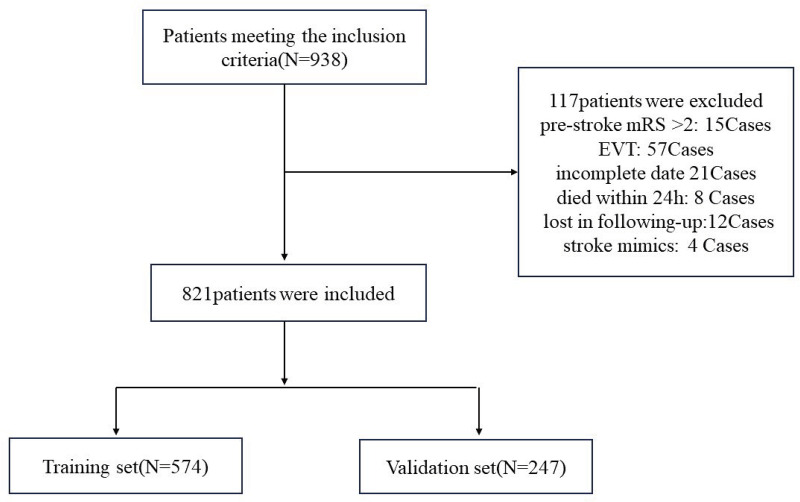
Study flowchart.

During the study period from January 2017 to February 2023, 938 patients met the inclusion criteria. The 117 patients who met the exclusion criteria were subsequently excluded from the study, leaving 821 patients eligible for data analysis, including 574 in the training group and 247 in the validation group (Fig 1).

### Variable selection

Table 1 shows a comparison of the baseline characteristics of the two groups of patients included in the study. 48 potential indicators associated with poor prognosis at 6 months after intravenous thrombolysis were included in this study:

**Table 1:** Comparison of baseline information for training and validation sets.

Variables	Total (n = 821)	Training set (N = 574)	Validation set (N = 247)	P value
Age, y Median (Q1, Q3)	65 (56, 71)	65 (56, 72)	64 (54.5, 71)	0.101
Gender, n (%)				0.113
Female	277 (34)	204 (36)	73 (30)	
Male	544 (66)	370 (64)	174 (70)	
BMI, Median (Q1, Q3)	24.9 (22.23, 27.04)	24.77 (22.23, 26.99)	25.2 (22.36, 27.28)	0.388
anterior/ posterior circulation n (%)				0.895
Anterior	629 (77)	441 (77)	188 (76)	
Posterior	192 (23)	133 (23)	59 (24)	
TOAST, n (%)				0.398
LAA	641 (78.1)	443 (77.2)	198 (80.2)	
SAO	109 (13.3)	82(14.3)	27(11.0)	
CE	59 (7.2)	40 (7.0)	19 (7.7)	
SOE	6 (0.7)	4(0.7)	2 (0.8)	
SUE	6(0.7)	5(0.8)	1 (0.3)	
SBP, Median (Q1, Q3)	146 (135, 159)	146 (135, 159)	146 (133.5, 158.5)	0.499
DBP, Median (Q1, Q3)	82 (76, 89)	81.5 (76, 89)	83 (76.5, 90)	0.130
ANIHSS, Median (Q1, Q3)	5 (2, 11)	5 (2, 11)	4 (2, 10.5)	0.440
Post thrombolysis, NIHSS Median (Q1, Q3)	3 (1, 10)	3 (1, 10)	3 (1, 9)	0.453
OTT, min, Median (Q1, Q3)	128 (91, 175)	127.5 (90.25, 176)	128 (92.5, 169.5)	0.891
DNT, min Median (Q1, Q3)	48 (35, 73)	50 (34, 77)	46 (35, 67)	0.249
Smoking, n (%)				0.034
*No*	440 (54)	322 (56)	118 (48)	
*Yes*	381 (46)	252 (44)	129 (52)	
Drinking, n (%)				0.354
*No*	559 (68)	397 (69)	162 (66)	
*Yes*	262 (32)	177 (31)	85 (34)	
Diabetes, n (%)				0.170
*No*	635 (77)	452 (79)	183 (74)	
*Yes*	186 (23)	122 (21)	64 (26)	
Hypertension, n (%)				0.577
*No*	316 (38)	225 (39)	91 (37)	
*Yes*	505 (62)	349 (61)	156 (63)	
Hyperlipidemia, n (%)				0.074
*No*	811 (99)	570 (99)	241 (98)	
*Yes*	10 (1)	4 (1)	6 (2)	
Atrial fibrillation, n (%)				0.990
*No*	721 (88)	504 (88)	217 (88)	
*Yes*	100 (12)	70 (12)	30 (12)	
HCY, n (%)				0.175
*No*	542 (66)	370 (64)	172 (70)	
*Yes*	279 (34)	204 (36)	75 (30)	
Previous stroke, n (%)				0.320
*No*	708 (86)	500 (87)	208 (84)	
*Yes*	113 (14)	74 (13)	39 (16)	
CHD, n (%)				0.679
*No*	639 (78)	444 (77)	195 (79)	
*Yes*	182 (22)	130 (23)	52 (21)	
WBC counts ×10^9^/L, Median (Q1, Q3)	7.37 (5.93, 9.06)	7.33 (5.86, 8.98)	7.39 (6.09, 9.21)	0.388
HB, g/L Mean ± SD	144 (133, 155)	144 (132, 155)	144 (133, 157)	0.309
PLTconut×10^9^/L, Median (Q1, Q3)	216 (179, 256)	216.5 (174, 255)	216 (183, 259)	0.415
Ncount×10^9^/L, Median (Q1, Q3)	4.47 (3.41, 5.92)	4.46 (3.39, 5.92)	4.51 (3.5, 5.86)	0.770
Mcount×10^9^/L, Median (Q1, Q3)	0.51 (0.4, 0.64)	0.51 (0.39, 0.64)	0.51 (0.41, 0.64)	0.936
L%, Median (Q1, Q3)	1.86 (1.35, 2.5)	1.83 (1.34, 2.48)	1.87 (1.38, 2.52)	0.249
NLR, ratio Median (Q1, Q3)	2.26 (1.5, 3.87)	2.35 (1.51, 3.94)	2.18 (1.49, 3.66)	0.447
MNR, ratio Median (Q1, Q3)	0.11 (0.08, 0.15)	0.11 (0.08, 0.15)	0.11 (0.08, 0.15)	0.831
Ecount×10^9^/L, Median (Q1,Q3)	0.1 (0.05, 0.17)	0.1 (0.04, 0.16)	0.1 (0.05, 0.18)	0.272
PNR, ratio Median (Q1, Q3)	47.47 (35.58, 64.69)	47.52 (35.34, 64.2)	47.37 (35.85, 65.01)	0.909
NER, ratio Median (Q1, Q3)	46.3 (24.2, 102.6)	46.14 (25.1, 106.34)	46.43 (22.66, 90.77)	0.362
EMR, ratio Median (Q1, Q3)	0.2 (0.1, 0.33)	0.19 (0.09, 0.32)	0.21 (0.11, 0.35)	0.272
PT, s, Median (Q1, Q3)	10.8 (10.2, 11.5)	10.8 (10.22, 11.5)	10.7 (10.2, 11.5)	0.367
APTT, s Median (Q1, Q3)	26.4 (24.6, 28.8)	26.4 (24.6, 28.78)	26.4 (24.75, 28.75)	0.380
INR2, Median (Q1,Q3)	0.93 (0.88, 0.98)	0.93 (0.88, 0.99)	0.92 (0.88, 0.97)	0.280
GLU, μ mol/L Median (Q1,Q3)	6.88 (5.84, 8.9)	6.82 (5.83, 8.65)	7.01 (5.86, 10.09)	0.361
BNP, p g/ml Median (Q1, Q3)	28.3 (11.2, 113)	32 (12, 121.75)	25.5 (9.3, 92.9)	0.153
LDH, U/L Median (Q1, Q3)	383.13 (208, 480)	388.13 (210, 490)	375.47 (208, 450.21)	0.150
Cr, μ mol/L Median (Q1,Q3)	67 (57.1, 79)	66.8 (57.15, 78.29)	67.69 (57.05, 79.6)	0.313
UA, μ mol/L Median (Q1,Q3)	323.51 (263.4, 393.84)	322.3 (262.3, 393.28)	327.8 (266.25, 394.59)	0.650
TC, mmol/L Median (Q1, Q3)	4.56 (3.87, 5.31)	4.58 (3.92, 5.36)	4.52 (3.75, 5.14)	0.062
TG, mmol/L Median (Q1, Q3)	1.27 (0.92, 1.78)	1.3 (0.92, 1.8)	1.24 (0.91, 1.73)	0.278
HDL, mmol/L Median (Q1, Q3)	1.03 (0.82, 1.23)	1.02 (0.82, 1.24)	1.03 (0.84, 1.23)	0.815
LDL, mmol/L Median (Q1, Q3)	2.7 (2.1, 3.28)	2.72 (2.12, 3.36)	2.6 (1.99, 3.18)	0.110
HDL/LDL, ratio Median (Q1, Q3	0.37 (0.29, 0.49)	0.37 (0.29, 0.48)	0.39 (0.31, 0.52)	0.089
MHR, ratio Median (Q1, Q3)	0.5 (0.36, 0.74)	0.5 (0.36, 0.74)	0.5 (0.35, 0.76)	0.944

**Fig 2:**
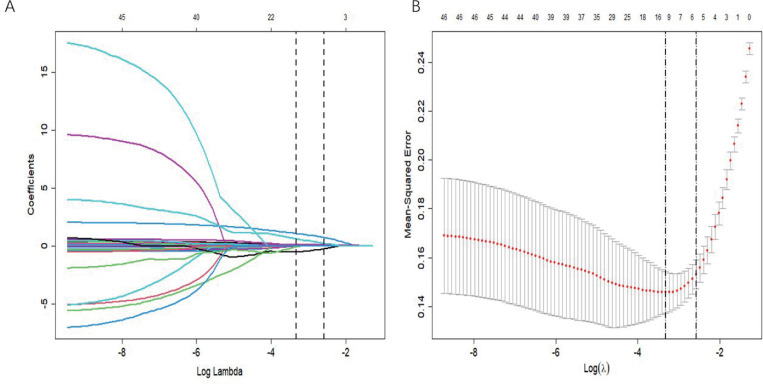
LASSO regression analysis. **(A)** In LASSO regression coefficient profiles, individual lines represent different predictor variables as the regularization parameter (lambda) increases. **(B)** LASSO regression Lambda Selection.

In order to avoid overfitting and improve model accuracy, we used LASSO regression technique and glmnet package implementation technique in R.10 (Fig 2A, B) for cross-validation to determine the optimal regularization parameter (λ = 0.07566604). We performed penalty coefficient compression to reach seven key variables viz: DNT, HCY, ANIHSS, PNIHSS, LDH, HDL, H/L, and MHR (Table 2).

**Table 2:** Coefficients and lambda.1SE value of the LASSO regression.

Variable. Variable	Variable. Coefficient	Lambda.1SE
DNT	0.0044025195	0.07566604
HCY	1.0913086816	
LDH	0.0006396725	
HDL	−0.5042187117	
ANIHSS	0.0503328864	
PNIHSS	0.1256410000	
H/L	−0.0546661234	
MHR	0.6541324739	

### Multivariate analysis

Incorporating the above seven variables into a logistic regression resulted in the five indicators most associated with poor 6-month prognostic outcomes.

Multivariate regression analysis was subsequently employed to demonstrate that the variable DNT [odds ratio (OR), 1.102; 95% confidence interval (CI), 1.005–1.019; p = 0.001], HCY(OR, 5.306; 95% CI,3.272–8.738; p < 0.001), LDH (OR, 1.002; 95% CI,1.000–1.003; p = 0.007), PNIHSS (OR, 1.267; 95% CI, 1.209–1.334; p < 0.001)and MHR (OR, 10.549; 95% CI, 4.798–24.57; p < 0.001) served as a significant predictor of unfavorable prognosis (Table 3).

**Table 3:** Independent risk factors associated with 6-month poor outcomes after intravenous thrombolysis in the training cohort.

Variable	B	SE	OR	CI	Z	P
DNT	0.012	0.00366	1.012	1.012 (1.005-1.019)	3.343	0.001
HCY	1.669	0.25004	5.306	5.306 (3.272-8.738)	6.675	<0.001
LDH	0.002	0.00076	1.002	1.002 (1.000-1.003)	2.715	0.007
PNIHSS	0.237	0.02503	1.268	1.267 (1.209-1.334)	9.487	<0.001
MHR	2.356	0.41569	10.549	9.54 (4.798-24.57)	5.668	<0.001

### Predictive model development

**Fig 3:**
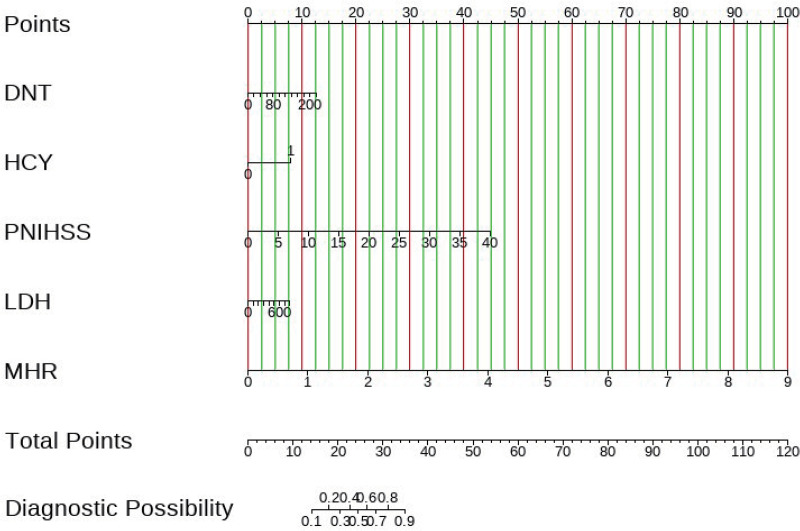
The Nomogram for predicting the 6-month unfavorable outcome.

Multivariate logistic regression analysis of acute stroke patients identified five predictors of poor prognosis: DNT, HCY, LDH, PNIHSS and MHR. Nomograms were constructed using these variables, and point values were assigned to each variable. The cumulative score of these five predictors helped to estimate the likelihood of poor prognosis at 6 months after intravenous thrombolysis in patients with acute ischaemic stroke (Fig 3).

Example: A patient with a DNT of 80 minutes prior to thrombolysis – corresponds to a score of 5 on the nomogram, a history of HCY denial – corresponds to a score of 0 on the nomogram, an LDH of 20 U/L – corresponds to a score of 1 on the nomogram, a pre-thrombolysis NIHSS of 2 – corresponds to a score of 1 on the nomogram, and an MHR of 1 – corresponds to a score of 11 on the nomogram, giving the patient a total score of 18. The probability of having an unfavorable prognosis at 6 months after thrombolysis is 0.2 on the nomogram (red dashed line S1 Fig. nomogram), i.e., there is a twenty percent chance that the patient will have an unfavorable prognosis, the benefits of thrombolysis outweigh the drawbacks, and the patient is suitable for thrombolysis. Clinicians can instruct patients to actively pursue thrombolysis.

The discriminative performance of the model was assessed by calculating the AUC-ROC of the training cohort (AUC, 0.914; 95% CI, 0.899– 0.939). The model was externally validated using the AUC-ROC of the test cohort (AUC, 0.892; 95% CI, 0.852–0.932).

**Fig 4:**
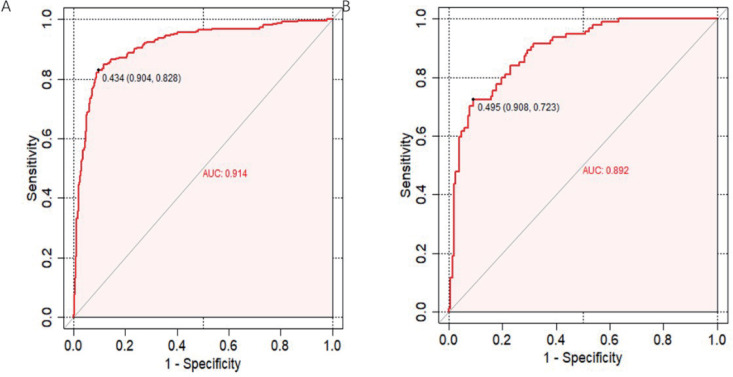
Receiver operating characteristic curves. **(A)** Receiver operating characteristic (ROC) curves for the predictive model in the training dataset. **(B)** Receptor Action Characterization (ROC) Curve for Predictive Models in the Validation Dataset.

Upon validation of the nomogram, it was determined that high levels of sensitivity and specificity were evident in both the training and validation sets. This indicates that the model possesses the ability to accurately identify true positive and true negative cases with unfavorable prognoses. Respectively: training set: cut-off of 0.434, sensitivity of 0.828, specificity of 0.904; validation set; cut-off of 0.495, sensitivity of 0.723, specificity of 0.908. (Figs 4A, B).

**Fig 5:**
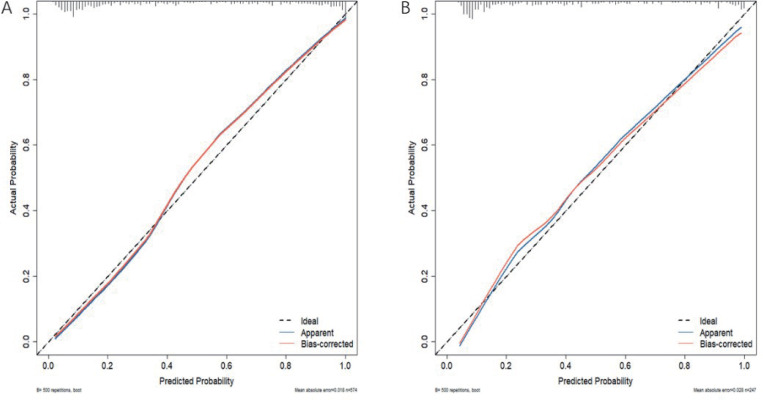
Calibration plot of the nomogram. **(A)** Calibration plot of the nomogram in the training cohorts and **(B)** Calibration plot of the nomogram in the training cohorts in the validation cohorts.

The calibration curves of the training and validation nomograms were close to the ideal slope value of 1.00, indicating that the calibration curves of the predictive model fit well to the ideal curves in the modelling and validation cohorts. It was consistent with the actual probability that the predictive model predicted a poor prognosis (Figs 5A, B).

**Fig 6:**
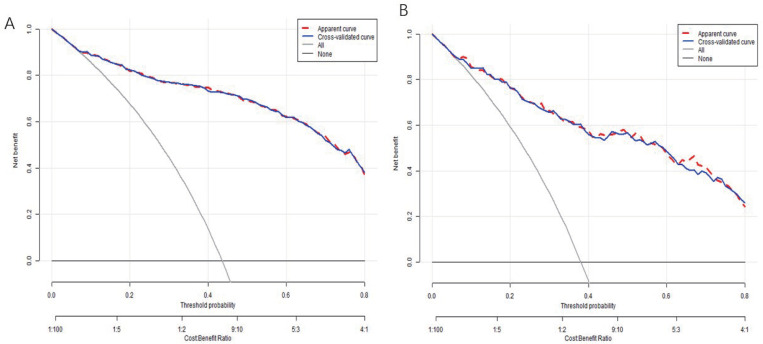
Decision curve analysis. **(A)** Decision curve analysis of the nomogram of training cohort and **(B)** Decision curve analysis of the nomogram of validation cohort.

A decision curve analysis (DCA) was performed to assess the prognostic utility of the existing prediction model. This analysis measured the net benefit by comparing the true and false-positive results, revealing that the current nomogram yielded a greater net benefit compared to treatment based solely on the NIHSS (Figs 6A, B).

**Fig 7:**
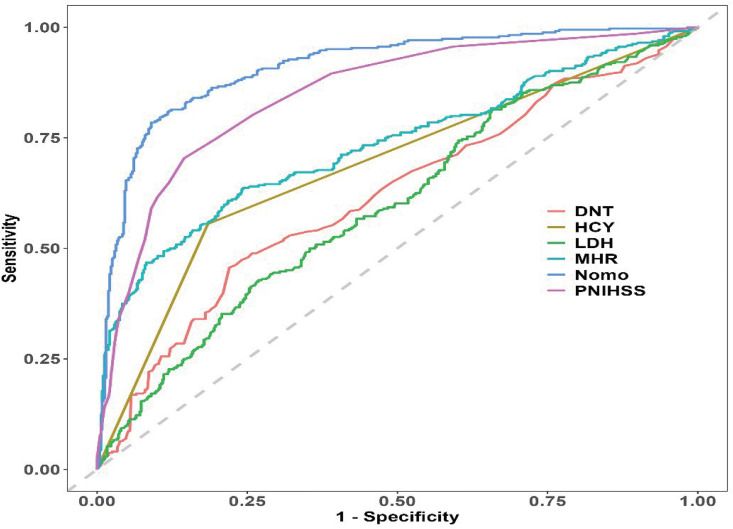
A reasonableness analysis was made based on model probabilities. It was shown that the area under the curve of the constructed model was greater than the area under the curve of the model constructed for any single factor in the model.

Rationality validation of the model was then performed, which showed that the Receiver Operating Characteristic (ROC) of the 821-based nomogram showed a statistically significant increase over that of the single-variable nomogram, indicating clear rationality (Fig 7).

**Fig 8:**
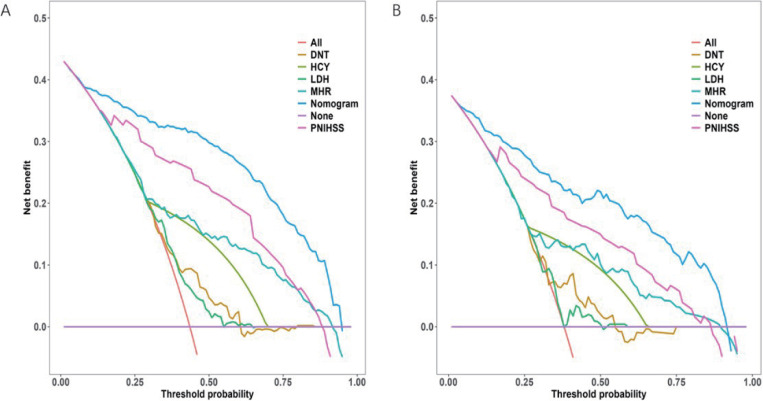
Comparison of the Decision curve analysis. Comparison of the Decision curve analysis (DCA) of the constructed model with the decision curves of each variable individually in the model in the training (A) and validation (B) sets.

A comparison is also made between the decision curve analysis (DCA) of the visual representation and the decision curves of the model for each variable in both the training and validation sets. This comparison highlights the significant clinical utility of the constructed nomograms, as shown in Fig 8 (A, B).

## Discussion

This study primarily identified that the five factors—door-to-needle time (DNT), homocysteine (HCY), lactate dehydrogenase (LDH), post-thrombolysis National Institutes of Health Stroke Scale (NIHSS) score, and monocyte-to-high-density lipoprotein ratio (MHR)—are instrumental in constructing nomograms that can predict poor prognosis in patients with acute ischemic stroke six months following intravenous thrombolysis. These findings furnish clinicians with a valuable tool for early prognostic assessment, facilitating timely intervention and management of adverse factors.

The predictors included in the model were derived from routine clinical examinations and were readily available. These variables have previously been reported to be associated with poor outcomes in patients with AIS. The NIHSS score [14] is widely recognised as a valuable tool for assessing stroke severity in patients. The pre-thrombolysis NIHSS score reflected the baseline status of the patients, and the post-thrombolysis NIHSS score reflected the ischaemia-reperfusion status of the patients. This distinction helps to differentiate patients who are clinically effective from those who are not, or who do not have reperfusion, and improves the ability to predict clinical and functional outcomes [15]. A recent study has also shown that the use of the NIHSS [16] score to assess clinical outcome can facilitate a more precise examination of structure-function relationships, thereby improving contemporary clinical assessment of stroke and the development of predictive models of stroke outcome in the field of neuroimaging.

Door-to-needle time (DNT) [17] is a key factor in the emergency treatment of AIS, and the implementation of in-hospital stroke systems has significantly reduced DNT and ultimately improved patient outcomes. Shumel et al [18] showed that in patients aged 65 years and older receiving intravenous thrombolytic therapy, shorter DNT was associated with lower all-cause mortality and lower one-year all-cause readmission rates. These results highlight the importance of efforts to reduce the duration of thrombolytic therapy. The shorter the DNT [19], the less likely acute stroke patients with a poor prognosis were to be discharged from hospital and the greater the benefit of thrombolysis.

Homocysteine (HCY) [20] is a sulphur-containing amino acid and is the only direct precursor of L-methionine biosynthesis, a process that depends on the presence of vitamin B₁₂. This pathway is part of the “one carbon” unit metabolism. The catabolism of HCY requires the presence of vitamin B₆, so any disturbance in folate and B vitamin levels will hinder the conversion of HCY. Several studies [21,22] have shown that high levels of homocysteine (HCY) are an independent risk factor for cardiovascular and cerebrovascular disease, with adverse effects on the vascular endothelium and cerebral vessels. Plasma HCY levels can be used as a prognostic indicator of the risk of death in patients with AIS. After thrombolysis, the higher the HCY level, the worse the clinical prognosis and the higher the mortality. Lactate dehydrogenase (LDH) is a type of glycolytic enzyme that is widely distributed in human tissues. LDH is an important contributor to glucose metabolism and its release into the blood indicates changes in membrane integrity, function and permeability following tissue injury. In acute stroke [23], elevated levels of lactate dehydrogenase are associated with ischaemia-induced brain cell damage. Monitoring lactate dehydrogenase levels shows promise in predicting prognosis in patients with acute stroke. Jin et al [24] found that elevated serum LDH levels predicted adverse clinical outcomes after intravenous thrombolysis in patients with acute ischaemic stroke, which is consistent with the current findings.

Atherosclerotic oxidation has been identified as a major cause of stroke, with significant involvement of blood monocytes. Monocytes [25], after differentiation into macrophages, have different pro-inflammatory or anti-inflammatory properties depending on their environment. Macrophages and smooth muscle cells secrete pro-inflammatory cytokines such as IL-1β, IL-12 and IL-6, leading to a chronic inflammatory state that eventually results in the degradation of the fibrous cap and the formation of thin-cap atherosclerotic plaques that are prone to rupture. After the onset of AIS [26,27], cerebral ischaemia and hypoxia stimulate monocytes to produce a variety of inflammatory mediators that can activate platelets and trigger a series of reactions that exacerbate the damage to ischaemic brain cells. HDL [28], also known as high-density lipoprotein, plays a crucial role in the reverse transport of cholesterol and has several protective functions in the development of atherosclerosis. HDL molecules are involved in the regulation of monocyte activation, adhesion and macrophage migration, as well as the control of progenitor cell proliferation and differentiation into monocytes. In addition, HDL [29] has anti-inflammatory properties in macrophages or fat cells by removing potentially harmful lipid hydroperoxides from the blood. HDL also promotes endothelial repair and has antithrombotic effects by increasing the availability of nitric oxide in blood vessels. Therefore, the monocyte/high-density lipoprotein cholesterol ratio (MHR) [30] is emerging as a new systemic indicator of atherosclerosis-associated inflammation with potential prognostic value in cardiovascular disease. Monocytes have pro-inflammatory properties, while HDL cholesterol plays a protective role. Elevated MHR has been shown to independently predict all-cause mortality and poor functional outcome in patients with ischaemic stroke or transient ischaemic attack (TIA), suggesting that it has the potential to serve as an important and independent predictor of poor functional outcome in patients with AIS. The present study provides further evidence that the MHR [31] may be a prognostic indicator for intravenous thrombolytic therapy in patients with acute stroke. The present study provides further evidence that the MHR [31] may be a prognostic indicator for intravenous thrombolytic therapy in patients with acute stroke. Multivariate logistic analysis was used to control for confounding variables and PNIHSS score and MHR were found to be significant contributors to the final prediction model.

AIS is characterised by increased rates of morbidity, disability, mortality and recurrence [32]. Timely evaluation of patients with poor prognosis, timely intervention, improve the long-term survival rate of patients, it is urgent. Our research has several advantages. Firstly, the inclusion of patients with a wide range of clinical data variables allows for a more comprehensive collection of information and the use of powerful databases. In addition, the implementation of the LASSO regression technique helps to prevent overfitting of the model, thereby improving the usefulness of the model and facilitating the development of intuitive predictive models for the assessment of poor outcomes in patients undergoing acute ischaemic venous thrombolysis. This method is more suitable for clinical use and is useful for early identification of poor prognosis after thrombolysis in ischaemic stroke patients. More importantly, our study was an evaluation of the long-term prognosis after 6 months of intravenous thrombolysis in patients with acute ischaemic stroke. Compared with previous studies, this further confirms that the AUC value of the training set of the line graph for predicting poor prognosis after 6 months of intravenous thrombolysis is greater than that of the 3-month prognosis (0.914 * 0.870), and the validation set is also larger (0.892 * 0.822) [33]. The longer duration model has a higher ability to distinguish poor prognosis after intravenous thrombolysis. At this point in time, the prognosis of patients has a significant impact on their overall prognosis. Clinicians can effectively identify high-risk patients early through visual mapping, communicate prognosis to patients and their families in a timely manner, formulate tailored treatment and rehabilitation plans, and ultimately reduce the likelihood of poor prognosis, reduce disability and optimise the allocation of social resources.

The study has a number of limitations. First, the data used in this study came from a single-centre database and could be considered limited in scope. Future studies are recommended to extend and validate these findings using a multicentre approach. Second, certain variables known to be associated with AIS outcomes, such as age [34], hypertension [35], and time from onset to treatment [36], were not observed in this study and warrant further investigation. Finally, the retrospective nature of the analysis may introduce biases, such as information bias, which should be recognised and addressed in future studies.

## Conclusion

A dependable visual nomogram incorporating DNT, HCY, LDH, PNIHSS, and MHR can effectively predict the poor prognosis of patients with acute ischemic stroke (AIS) undergoing intravenous thrombolytic therapy with alteplase over a six-month period. This tool facilitates the early identification of patients who may benefit from supplementary intensive interventions, mitigates complications associated with intravenous thrombolysis for stroke, enhances patients’ quality of life, and optimizes the allocation of healthcare and social resources.

## Supporting information

S1 Fig Application example of nomogram. (EPS)

S1 File Data. (CSV)
